# Long-Term Endurance and Power Training May Facilitate Motor Unit Size Expansion to Compensate for Declining Motor Unit Numbers in Older Age

**DOI:** 10.3389/fphys.2019.00449

**Published:** 2019-04-26

**Authors:** M. Piasecki, A. Ireland, J. Piasecki, H. Degens, D. W. Stashuk, A. Swiecicka, M. K. Rutter, D. A. Jones, J. S. McPhee

**Affiliations:** ^1^Clinical, Metabolic and Molecular Physiology, MRC-ARUK Centre for Musculoskeletal Ageing Research, National Institute for Health Research (NIHR) Nottingham Biomedical Research Centre, University of Nottingham, Nottingham, United Kingdom; ^2^School of Healthcare Science, Manchester Metropolitan University, Manchester, United Kingdom; ^3^Musculoskeletal Physiology Research Group, Sport, Health and Performance Enhancement Research Centre, School of Science and Technology, Nottingham Trent University, Nottingham, United Kingdom; ^4^Institute of Sport Science and Innovations, Lithuanian Sports University, Kaunas, Lithuania; ^5^Department of Systems Design Engineering, University of Waterloo, Waterloo, ON, Canada; ^6^Division of Diabetes, Endocrinology and Gastroenterology, School of Medical Sciences, Faculty of Biology, Medicine and Health, The University of Manchester, Manchester, United Kingdom; ^7^Manchester Diabetes Centre, Central Manchester University Hospitals NHS Foundation Trust, Manchester Academic Health Science Centre, Manchester, United Kingdom; ^8^Department of Sport and Exercise Sciences, Musculoskeletal Science and Sports Medicine Research Centre, Manchester Metropolitan University, Manchester, United Kingdom; ^9^Department of Physiology, University of Padova, Padova, Italy

**Keywords:** exercise, motor unit, muscle, master athlete, electromyogarphy

## Abstract

The evidence concerning the effects of exercise in older age on motor unit (MU) numbers, muscle fiber denervation and reinnervation cycles is inconclusive and it remains unknown whether any effects are dependent on the type of exercise undertaken or are localized to highly used muscles. MU characteristics of the vastus lateralis (VL) were assessed using surface and intramuscular electromyography in eighty-five participants, divided into sub groups based on age (young, old) and athletic discipline (control, endurance, power). In a separate study of the biceps brachii (BB), the same characteristics were compared in the favored and non-favored arms in eleven masters tennis players. Muscle size was assessed using MRI and ultrasound. In the VL, the CSA was greater in young compared to old, and power athletes had the largest CSA within their age groups. Motor unit potential (MUP) size was larger in all old compared to young (*p* < 0.001), with interaction contrasts showing this age-related difference was greater for endurance and power athletes than controls, and MUP size was greater in old athletes compared to old controls. In the BB, thickness did not differ between favored and non-favored arms (*p* = 0.575), but MUP size was larger in the favored arm (*p* < 0.001). Long-term athletic training does not prevent age-related loss of muscle size in the VL or BB, regardless of athletic discipline, but may facilitate more successful axonal sprouting and reinnervation of denervated fibers. These effects may be localized to muscles most involved in the exercise.

## Introduction

Voluntary movements are controlled through precise activation of motor unit (MU) populations. A single MU is composed of an alpha motor neuron with its cell body in the ventral horn of the spinal cord, an axon, and all of the muscle fibers innervated by that single motor neuron. Conditions leading to declining MU numbers and alterations of MU sizes, such as advancing older age, may impact upon many aspects of neuromuscular function and physical performance. Estimates made from cadaveric specimens showed around 30% fewer motor neuron cell bodies in the lumbo-sacral spinal cord of older compared with younger adults (Kawamura et al., 1977; Tomlinson and Irving, 1977; Mittal and Logmani, 1987). Electrophysiological techniques using surface and intramuscular electromyography (EMG) also report 30–50% fewer MUs by the age of 70 years in human limb muscles (McNeil et al., 2005; Piasecki et al., 2016a,c). Whilst the age-related reductions in neuromuscular control are multifactorial including alterations in MU firing rate (Dideriksen et al., 2012), proprioception and afferent feedback (Baudry, 2016), the lower MU numbers observed in older age directly coincides with loss of muscle fibers (Lexell et al., 1988; McPhee et al., 2018) and contributes to sarcopenia defined by low muscle mass and functional impairments.

We have shown that the loss of MUs occurs relatively early into older age and precedes sarcopenia (Piasecki et al., 2016c) because not all of the muscle fibers of affected MUs are lost; some may be “rescued” by axonal sprouting of adjacent surviving neurons, thereby helping to preserve total muscle mass. As a consequence, older muscle has fewer MUs but they are larger in size (Hepple and Rice, 2016; Piasecki et al., 2016b). The relative success of sprouting may differ between people and influence the rate of muscle declines during aging. For example, the reinnervation process may be less successful in sarcopenic individuals with the smallest muscle mass in older age (Piasecki et al., 2018c). Thus, finding an intervention to minimize motor neuron loss and associated muscle fiber denervation, and/or increasing axonal sprouting to rescue denervated fibers would preserve muscle mass and function for longer into older age. Regular intense exercise is one possible intervention.

There is conflicting evidence concerning the potential benefits of regular intense exercise to preserve MU numbers and enhance axonal sprouting. One cross-sectional study showed higher MU numbers in the tibialis anterior (TA) of athletic old compared with non-athletic age-matched controls (Power et al., 2010), but MU numbers of the biceps brachii (BB) were not maintained (Power et al., 2012), suggesting these effects are not systemic, but are localized to the highly used muscles. However, a similar cross-sectional study showed no such preservation of MU numbers in the TA of older runners (Piasecki et al., 2016a). Previous studies in this field have lacked useful comparison between groups by failing to include highly athletic young as comparators for the athletic old and not distinguishing power or endurance athletes (Power et al., 2010; Piasecki et al., 2016a). This is a problem because it is not known whether high performance younger athletes have different MU numbers or sizes to non-athletic young, or if one discipline may differ from another. Therefore, when investigating associations between aging, exercise and MU characteristics, it is more appropriate to include athletic old and athletic young adults competing in endurance and power disciplines, along with age-matched controls. An alternative approach is to compare muscles within the same person after long-term discordance of exercise exposure between limbs, as occurs with racquet sports such as tennis.

The aims of the present study were to determine whether estimated MU numbers and motor unit potential (MUP) sizes differ between young power and endurance athletes, and whether decades of exercise into older age preserves MU numbers and increases MUP size. Three main hypotheses were tested. Firstly, that masters athletes would have *fewer* vastus lateralis (VL) MUs than young athletes and similar to age-matched non-athletes. This would indicate that exercise training does not prevent typical age-related MU loss. Secondly, that masters athletes would have *larger* MUPs than young athletes and age-matched non-athletes. This would indicate that exercise enhances reinnervation of denervated fibers to increase MU size. Finally, to account for any possibility of secular changes affecting MU numbers or sizes in cross-sectional studies and to examine the notion of localized influences on MU plasticity, we hypothesized that the favored racquet arm of masters tennis players would have similar MU numbers, but larger MUP sizes compared with the non-favored arm.

## Materials and Methods

### Participants and Ethical Approval

The study was conducted in accordance with the *Declaration of Helsinki* and approved by the Manchester Metropolitan University Research Ethics Committee and the National Research Ethics Service Committee Northwest (15/NW/0426). All participants provided written informed consent. Eighty-five young and older males participated in the first part of this study. This included 15 young controls, 8 young endurance athletes, 11 young power athletes, 22 older controls, 18 master endurance athletes and 11 master power athletes. Eleven older male tennis players took part in the second part of the study. The young and old controls were recruited from the University population and the local community; they were recreationally active but did not compete in any sports. All athletes (young and old) were recruited from running clubs, two national masters athletics competitions and from an advertisement placed in a national athletics magazine.

For part two of the study, masters tennis players were recruited from the British Seniors’ Indoor Tennis Championships and tested onsite at the competition location.

### Athlete Status

Part 1: All of the athletes were actively competing in their respective sports and distances at the time of testing and all completed more than 5 h of training per week specific to their sport or discipline. All of the master athletes had trained specifically for their events since young adulthood (>18 years) and the median number of years of training at the point of testing was 46 years for the endurance athletes and 51 years for the power athletes. All young athletes had trained specifically for their respective events for a minimum of 5 years prior to testing. The age-graded performance (AGP) of an athlete allows a comparison of the athlete against the current world record within their age group and discipline. It is expressed as a percentage of the world record. All of the young endurance athletes competed at distances of 5000 m and above and had a mean AGP of 87.2 (2.1). The young power athletes consisted of a combination of competitive Olympic weightlifters and power lifters and had a mean AGP of 67.9 (3.4). The old endurance athletes all competed at distances of 3000 m and above; this group had a mean AGP of 79.1 (9.2) and included four athletes ranked in the top three in Great Britain for their respective ages and distances. The old power athletes all competed in sprinting distances of 400 m and below, and the mean AGP was 84.5 (9.9). This group included two athletes ranked in the top three in Great Britain for their respective ages and distances.

Part 2: Age-graded performances are not available for the masters tennis players. All athletes had trained and competed in tennis prior to the age of 50 years and were recruited and tested at a senior national competition.

### Anthropometric Assessments

**FIGURE 1:**
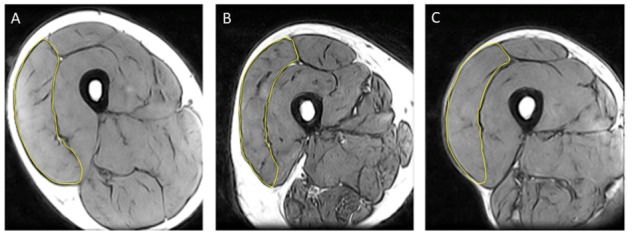
Representative MRI images highlighting vastus lateralis (VL) cross sectional area (CSA) from **(A)** young control; **(B)** older control; and **(C)** old endurance athlete.

Part 1: The cross-sectional area (CSA) of the VL at the motor point (approximately mid muscle belly) was measured in the right leg with magnetic resonance imaging (MRI) using a T1-weighted turbo 3D sequence on a 0.25-T G-Scan with the participants lying supine (Esaote, Genoa, Italy). The motor point was marked using an external reference taped to the skin that was a high-fat capsule and easily identifiable from an MRI scan. Contiguous transverse-plane slices of 6 mm thickness were collected. Images were exported and analyzed off-line as previously described, using Osirix imaging software (Osirix medical imaging, Osirix, Atlanta, GA, United States (Maden-Wilkinson et al., 2014; Figure 1). Body mass and height were measured using calibrated scales and stadiometry, respectively, and body mass index (BMI) calculated. Total body fat percentage was assessed by dual-energy X-ray absorptiometry (Lunar Prodigy Advance, version EnCore 10.50.086; GE Healthcare, Little Chalfont, United Kingdom) with the participant lying supine with legs and arms fully extended.

Part 2: The BB thickness was measured in both favored and non-favored arms using B-mode ultrasound (Esaote MyLab25, Genoa, Italy) with a 7.5 MHz linear probe held perpendicular to the BB muscle belly. The arm and forearm were relaxed and fully extended at 180°, supported at the shoulder and elbows, with the arm in the supinated position and palm of the hand facing anteriorly. The greatest distance from the superficial to the deep aponeurosis of BB was taken as the muscle thickness.

### Strength Assessments

Part 1: Right knee extensor strength was assessed with participants sitting with hips and knees flexed at 90° and the leg securely fastened to a force transducer 30 cm below the center of the knee joint. To familiarize with the equipment and to “warm-up” the muscle, participants performed a series of submaximal contractions. They were then instructed to perform a maximal isometric contraction, accompanied by verbal encouragement and visual feedback of force on a computer screen. This was repeated three times, with 60 s rest intervals. The best effort was taken as maximum voluntary isometric contraction force (MVC).

Part 2: In the masters tennis players, participants were seated with the shoulder abducted in the lateral plain to 90°. The height of the chair was adjusted so the participant could sit comfortably with the triceps resting on the base of the custom-built dynamometer. The elbow was flexed at 110° and the forearm was attached to the dynamometer around 27 cm from the tip of the elbow, with supination of the palm. Participants were familiarized to elbow flexion by performing three isometric contractions lasting 4 s at around 80% of maximal effort. Next, isometric elbow flexion MVC was assessed three times with 60 s rest between efforts. The highest value was accepted as MVC.

### Identifying the Motor Points

The motor point of the muscle is defined as the area of muscle providing the largest twitch from the smallest percutaneous stimulus (Botter et al., 2011). Motor points were identified using a cathode probe (Medserve, Daventry, United Kingdom) to apply percutaneous electrical stimulation at 400 V, pulse width of 50 μs, and current of around 8 mA, (DS7A Digitimer, Welwyn Garden City, Hertfordshire, United Kingdom). A self-adhesive electrode (Dermatrode, Farmadomo, NL) was used as the anode.

Part 1: The proximal motor point of the VL was located around 22 cm proximal to the lateral femoral condyle, close to the midline of the VL. The anode was placed over the right gluteus.

Part 2: The motor point of the BB was located around 14 cm from the medial epicondyle, and around 20 mm medially from the centreline of the two heads of the BB. The anode was placed over the acromion process of the scapula on the same side as the tested BB.

### Surface EMG

Part 1: The active recording sEMG electrode (disposable self-adhering Ag-AgCl electrodes; 95 mm^2^, Ambu Neuroline, Baltorpbakken, Ballerup, Denmark) was placed over the motor point and positioned to give the largest compound muscle action potential (CMAP, sometimes known as the M-wave) and shortest rise-time in response to stimulating the motor nerve. A reference electrode was placed over the patella tendon and a common ground electrode was placed over the patella for VL.

Part 2: For the BB, the active recording electrode was placed over the motor point, a reference electrode was placed over the lateral epicondyle and a common ground electrode was placed over the elbow.

In all cases the common ground electrode served for both surface and intramuscular EMG (iEMG) measurements. Surface EMG signals were bandpass filtered between 5 Hz and 5 kHz via CED 1902 amplifiers (Cambridge Electronics Design Ltd., Cambridge, United Kingdom). Signals were digitized with a CED Micro 1401 data acquisition unit (Cambridge Electronic Design). The sEMG signals were sampled at 5 kHz.

### Compound Muscle Action Potential

Part 1: Compound muscle action potentials were evoked using a manually triggered stimulator (model DS7AH; Digitimer). For the VL the anode was over the right gluteus, and the cathode probe over the femoral nerve, approximately half way between the anterior superior iliac spine and the pubic tubercle, proximal to the groin crease, but distal to the inguinal nerve.

Part 2: A bar electrode with the anode and cathode spaced 3 cm apart (Model MLADDF30; AD Instruments, Oxford, United Kingdom) was held over the musculotaneous nerve.

For both muscles the current was increased incrementally until the CMAP amplitude plateaued, generally between 100–200 mA. The current was then increased by 30 mA to ensure supra-maximal stimulation.

### Intramuscular EMG

After determining the MVC and CMAP, a concentric needle electrode (Model N53153; Teca, Hawthorne, NY) was inserted, immediately adjacent to the recording surface electrode over the motor point, to a depth of 1.5 – 2 cm into the VL or 1 – 1.5 cm into the BB. The iEMG signals were bandpass filtered from 10 to 10 kHz and sampled at 25 kHz. The force and EMG signals were displayed in real-time using Spike2 software (v8.01) and data were stored for off-line analysis.

### Recording From Individual Motor Units During Voluntary Contractions

The participant performed a voluntary, low force contraction while the needle position was adjusted to obtain intramuscular MUPs with peak second derivative values >5 kV/s^2^, thus ensuring the recording needle electrode was proximal to fibers belonging to the sampled MUs (Stashuk, 1999b). The participant then performed a voluntary contraction lasting 12–15 s, keeping as close as possible to a target line shown on the computer monitor that was set at 25% MVC with real-time visual feedback. The needle electrode was then repositioned by combinations of rotating the bevel 180° and withdrawing it by 2–5 mm. The procedure of needle positioning, voluntary contraction and signal recording was repeated until a minimum of six recordings from varying depths had been obtained to sample from representative sets of MUs. The participant rested for 30 s between contractions.

### EMG Analysis and Motor Unit Number Estimates

**FIGURE 2:**
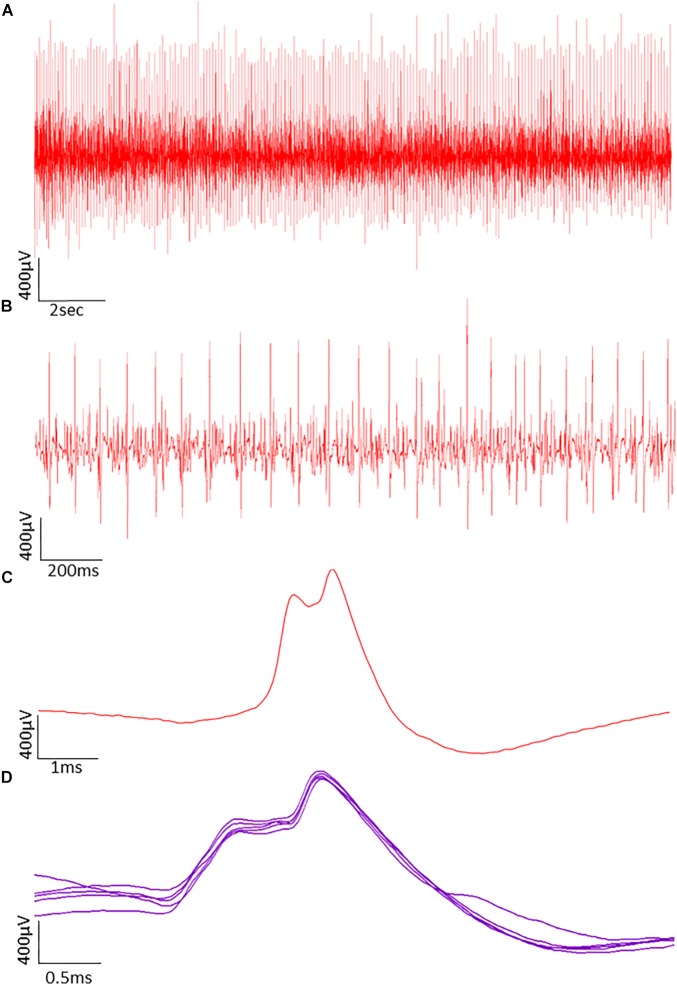
Typical raw data showing motor unit potentials (MUPs) recorded by intramuscular EMG. The MUPs were recorded from an older power athlete holding a sustained isometric contraction at 25% MVC. **(A)** MUPs from populations of active motor units. **(B)** Data from A shown with a reduced time window to reveal more detail. **(C)** A Single MUP isolated from the traces shown in B. **(D)** Traces of the same MUP overlaid from consecutive firing.

**FIGURE 3:**
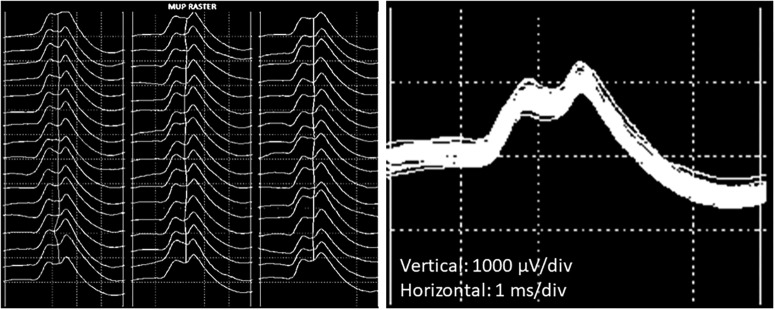
Example images of a single motor unit potential decomposed from intramuscular EMG recordings from the VL. (Left) Raster plot of a motor unit potential train (MUPT) showing 51 consecutive firings from the same MU. (Right) Shimmer plot with the MUPs from the left-hand image overlaid.

**FIGURE 4:**
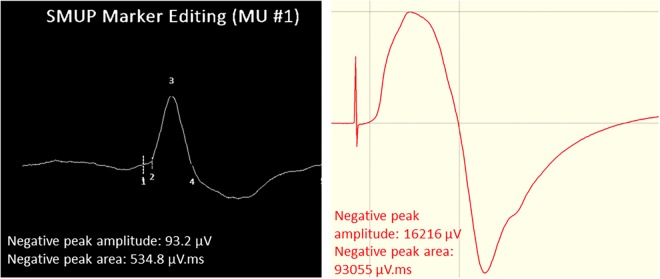
Example images of VL surface EMG measurements. Left: Surface motor unit potential (sMUP) averaged from 124 observations of the same MUP. Numbers indicate; 1, negative peak onset; 2, onset of intramuscular MUP; 3, negative peak amplitude; 4, negative peak end. (Right) Compound muscle action potential (CMAP) obtained from percutaneous electrical stimulation of the femoral nerve. Initial spike reflects the stimulus artifact.

The procedures for recording and analyzing individual MUPs and calculating motor unit number estimates (MUNE) values have been described in detail previously (Piasecki et al., 2016a,c). Briefly, intramuscular and surface EMG signals were analyzed using decomposition based quantitative electromyography (DQEMG) (Boe et al., 2005, 2006; Stashuk, 1999a) (see Figure 2, 3 for iEMG raw data). MUNE values were computed as the ratio of the size of the CMAP to the size of a mean surface MUP (sMUP). Surface MUPs provide a surface EMG-based representation of a sampled MU. A sMUP is calculated using ensemble-averaging of suitable segments of the surface EMG signal, identified using the MUPs from motor unit potential trains (MUPTs) of the separate MUs (Figure 3). MUPTs that had fewer than 40 MUPs were excluded. The mean number of MUPs contributing to make a corresponding sMUP here was 111 ( ± 25, range 60 – 336). The mean sMUP was obtained by ensemble-averaging all of the negative-peak-onset-aligned sMUPs of the MUs sampled in a muscle. For the VL, CMAP and sMUP size were quantified by the negative peak area. For the BB, 2 of the participants had excessive CMAP durations which artificially inflated CMAP area, therefore CMAP and sMUP size were represented using negative peak amplitude (Figure 4). Both area and amplitude values have been used in previous studies to represent CMAP and sMUP size and areas and amplitudes sampled from the same muscle are strongly correlated.

A MUNE value was calculated by dividing the CMAP area or amplitude by the area or amplitude of an ensemble-averaged mean sMUP. This method was developed for the study of small peripheral muscles where the entire muscle may be within the capture area of the surface electrode. However, this does not account for differences in muscle size therefore the intramuscular MUNE (iMUNE), calculated as MUP size normalized to muscle CSA, is also reported (Piasecki et al., 2018b). Values obtained by both methods are obtained from a single contraction intensity (25% MVC), and may be considered as an index which is positively correlated with the number of MUs within the muscle, but is not as an actual number of MUs (Piasecki et al., 2018b).

### Statistical Analysis

Differences between groups in part one of the study were evaluated using a two-way ANOVA (2 × 3 factorial design) with *age* and *discipline* as fixed factors. Where significant interactions between the effects of age and discipline were observed, interaction contrasts were performed to reveal the cause of the interaction. The contrast estimate represents the “difference between the differences”; in this case it shows how the age-related contrasts differ across the three disciplines. For example, comparing the difference between young and old *controls* with the difference between young and old *endurance* athletes is calculated as: difference between young and old controls *minus* the difference between young and old endurance athletes.

The interaction contrasts are displayed in brackets as a contrast estimate, 95% CI, *p*-value. Also, where a significant interaction was observed, *simple* main effects of *age* and simple main effects of *discipline* with pairwise comparisons and Bonferroni adjustments are reported. Where no significant interactions between the effects of *age* and *discipline* were observed, the main effects of *age* and *discipline* are reported. In part two of the study opposite arms was compared using a paired samples *t*-test. Data were log transformed when not normally distributed. Analysis was performed using SPSS Version 21 (SPSS, Chicago, IL) software and *p* ≤ 0.05 was considered statistically significant.

## Results

### Athlete Participant Characteristics

Eighty-five participants were recruited into six sub-groups according to age and exercise discipline. Participant characteristics are shown in Table 1. The only statistically significant interactions between the effects of age and discipline were for body mass (*p* < 0.001) and BMI (*p* = 0.001) due to the age-related contrasts (comparing young and old of the same discipline) being greater for power athletes compared to endurance or controls (both *p* < 0.001).

**Table 1:** Participant characteristics by age and exercise discipline.

	Young control (*n* = 15)	Young endurance (*n* = 8)	Young power (*n* = 11)	Old control (*n* = 22)	Old endurance (*n* = 18)	Old power (*n* = 11)	*P*-value for interaction	*P*-value for main effects between study groups	
							**Age × Discipline**	**Age difference**	**Discipline difference**

Age (yrs)	25.8 (4.9)	25.1 (4.3)	28.2 (3.2)	70.3 (3.6)	69.4 (3.9)	70.5 (6.7)	0.626	<**0**.**001**	0.1281
Height (m)	1.78 (0.07)	1.78 (0.04)	1.80 (0.05)	1.75 (0.06)	1.73 (0.06)	1.74 (0.07)	0.682	**0**.**002**	0.757
Body mass (kg)	74.7 (9.4)	65.7 (5.6)^a^	91.5(8.6)^a,b^	73.9 (6.8)	65.5 (6.9)^a^	74.6 (8.0)^b,c^	<**0**.**001**	**–**	**–**
BMI (kg/m^2^)	23.3 (2.7)	20.7 (1.3)^a^	28.2 (2.9)^a,b^	24.3 (1.9)	21.4 (1.6)^a^	24.7(2.5)^b,c^	**0**.**001**	–	**–**
Body Fat (%)	18.9 (7.5)	9.3 (3.9)	16.5 (7.9)	22.4 (5.3)	13.9 (5.5)	18.1 (5.9)	0.813	**0**.**037**	<**0**.**001**
Knee extensor MVC (N)	591 (148)	459 (159)	681 (110)	350 (99)	314 (83)	485 (52)	0.183	<**0**.**001**	<**0**.**001**


*Main effects of *age* and *discipline* are shown in final columns where there was no significant interaction between these effects. Where this interaction was significant, simple main effects of *discipline* are shown as: ^*a*^indicates difference to control within age group. ^*b*^indicates difference to endurance within age group. Simple main effects of *age* are shown as ^*c*^indicates young vs. old within-discipline difference. Abbreviations: m, meters; kg, kilograms, BMI, body mass index, ALM, appendicular lean mass, CSA, cross sectional area; MVC, maximal voluntary contraction. P-values for significant differences are shown in bold.*

Older men had a higher body fat % than younger men. Body fat % also differed significantly across disciplines, with controls having highest fat %, followed by power athletes and then endurance athletes with lowest. Older men had a lower knee extensor MVC than younger men. Knee extensor MVC also differed significantly across disciplines, as it was highest in power athletes, followed by controls, then endurance athletes (Table 1).

### Muscle Size

**FIGURE 5:**
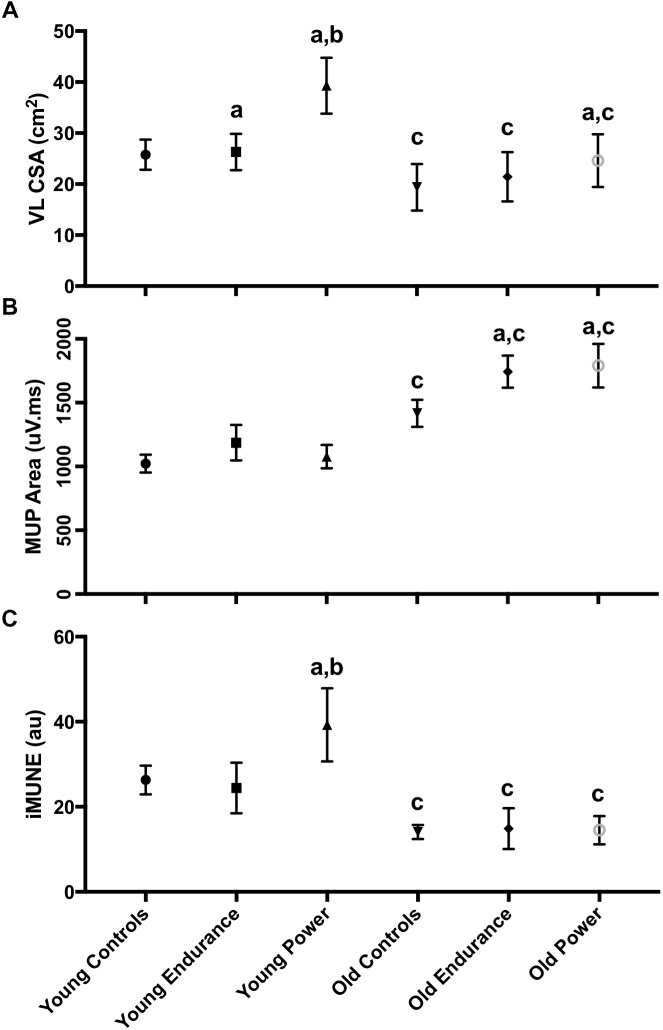
Neuromuscular characteristics of the VL. Error bars indicate 95% CI. VL iMUNE calculated as mean MUP normalized to VL CSA. Simple main effects: *a* indicates significant difference to control, within age group. *b* indicates significant difference to endurance, within age group. *c* indicates significant young vs. old within-discipline difference. Abbreviations: VL, vastus lateralis; CSA, cross sectional area; MUP, motor unit potential; iMUNE, intramuscular motor unit number estimate.

There was a significant interaction between the effects of age and discipline in VL CSA (Figure 5, *p* = 0.001 for interaction). Interaction contrasts revealed the age-related differences in VL CSA were greater in the power athletes than controls: the difference between young and old controls minus the difference between young and old power athletes was 8.28 cm^2^ (95% CI 3.29 to 13.26, *p* = 0.001); the difference between young and old controls minus the difference between young and old endurance athletes was 9.81 cm^2^ (95% CI 4.36 to 15.25, *p* = 0.001). There was no significant difference between values for young and old controls compared with young and old endurance athletes (1.53 cm^2^, 95% CI -3.23 to 6.29, *p* = 0.525). The VL CSA was larger in young than old in all three groups (all *p* < 0.001). VL CSA differed significantly across disciplines (*p* < 0.001) and was greater in the young compared with the older participants (*p* = 0.011). Pairwise comparisons showed the young controls did not differ from young endurance athletes (*p* > 0.99) but the young endurance and young controls had smaller VL CSA than young power (both *p* < 0.001). The old controls did not differ from old endurance (*p* = 0.494) but had significantly smaller VL CSA than old power (*p* = 0.009), while old endurance and old power did not differ significantly (*p* = 0.241) (Figure 5).

### Athlete CMAP, sMUP, and Estimates of MU Number

There were no statistically significant interactions between the effects of age and discipline on CMAP or sMUP area. The younger men had 22–50% larger CMAP area than old, with no difference across disciplines. The sMUP area did not differ by age or by discipline. As with CMAP, younger men had 18–38% higher MUNE values than the older men (*p* = 0.024), but MUNE values did not differ significantly across disciplines (*p* = 0.679) (Table 2). There was also a significant interaction between the effects of age and discipline in iMUNE (*p* = 0.002). Interaction contrasts revealed the age-related differences in iMUNE were significantly different in power athletes compared to age-related differences in controls (iMUNE contrast of differences: 12.52, 95% CI 4.57 to 20.47, *p* = 0.002) and age-related differences in endurance athletes (15.19, 95% CI 6.43 to 23.95, *p* = 0.001). There were no age-related differences between controls and endurance athletes (2.67, 95% CI -4.95 to 10.34, *p* = 0.096).

**Table 2:** Surface EMG measurements of the vastus lateralis.

	Young control (*n* = 15)	Young endurance (*n* = 8)	Young power (*n* = 11)	Old control (*n* = 22)	Old endurance (*n* = 18)	Old power (*n* = 11)	*P*-value for age × discipline interaction	*P*-value for age difference	*P*-value for discipline difference
CMAP (μV.ms)	98240 (20549)	92012 (14233)	98496 (24841)	65266 (15810)	74913 (19897)	70538 (10584)	0.263	**<0.001**	0.846
sMUP (μV.ms)	506 (234)	537 (201)	497 (223)	418 (145)	499 (178)	451 (215)	0.868	0.201	0.551
MUNE	233 (113)	199 (96)	219 (76)	168 (60)	168 (77)	184 (77)	0.687	**0.024**	0.679
MUs identified (number per person)	26 (12)	27 (12)	28 (11)	25 (9)	21 (10)	24 (11)	0.475	0.894	0.061


*Interaction effects and main effects of *age* and *discipline* are shown in final columns. CMAP, compound muscle action potential; sMUP, surface motor unit potential; MUNE, motor unit number estimate. P-values for significant differences are shown in bold.*

Intramuscular motor unit number estimates values were significantly larger in younger compared to older participants (*p* < 0.001) and pairwise comparisons showed this was consistent for all groups (controls *p* < 0.001; endurance *p* = 0.002; power *p* < 0.001). iMUNE values also differed across disciplines in the young (*p* < 0.001). Pairwise comparisons showed the iMUNE in young controls and young endurance were similar (*p* = 0.296), but young power had higher values than controls and endurance (*p* = 0.006 and *p* < 0.001, respectively). In older participants there was no significant effect of *discipline* on iMUNE (*p* = 0.946).

### Athlete Motor Unit Potential Size

There was a significant interaction between the effects of age and discipline for MUP area (Figure 5, *p* = 0.042 for interaction). Interaction contrasts showed the age-related increase in MUP size was smaller in the controls than it was for the endurance athletes (234 mV.ms, 95% CI 12 to 456, *p* = 0.038) and power athletes (314 mV.ms 95% CI 89 to 539, *p* = 0.013). There was no significant difference in the age-related contrast estimates in MUP size between endurance and power athletes (97 mV.ms, 95% CI -147 to 337, *p* = 0.292; Figure 5).

In younger participants, MUP area was smaller in controls, endurance athletes and power athletes compared with their discipline-matched older counterparts (all *p* < 0.001). MUP area in the young did not differ significantly across disciplines (*p* < 0.438), but MUP area did differ significantly across disciplines in the old (*p* < 0.001). Pairwise comparisons showed that older controls had smaller MUPs than older endurance athletes (*p* = 0.001) and older power athletes (*p* < 0.001), while MUP area in older endurance and older power athletes did not differ (*p* = 0.999; Figure 5).

### Masters Tennis Players

In the second part of the study, the BB of both arms (favored and non-favored) of masters tennis players (age: 74 ± 5.2 years) were compared. Of the 11 master tennis players included, 10 favored the right arm to hold the racquet and one favored the left arm. Muscle size assessed by thickness from ultrasound scanning did not differ significantly between arms, but strength was 7% greater in the favored arm (*p* = 0.044) (Table 3). The CMAP was 16% greater in the favored arm (*p* = 0.039), but there were no significant differences between arms in sMUP, MUNE or iMUNE values. MUPs recorded from the favored arm were around 20% larger than the non-favored arm (*p* < 0.001).

**Table 3:** Neuromuscular characteristics in the favored arm vs. the non-favored arm in masters tennis players.

	Favored arm *N* = 11	Non-favored arm *N* = 11	*P*-value
Muscle thickness (mm)	34.2 (4.8)	33.9 (4.7)	0.575
Elbow flexor MVC (N)	215 (68)	201 (70)	**0**.**044**
CMAP (μV)	13137 (4953)	11357 (4411)	**0**.**039**
sMUP (μV)	75.6 (26.9)	71.6 (42.1)	0.867
MUNE	194 (83)	163 (69)	0.188
iMUNE	31.0 (14.4)	31.6 (6.3)	0.979
MUP Area	1120 (709–1709)	934 (647–1430)	<**0**.**001**


*mm, millimeters; CMAP, compound muscle action potential; sMUP, surface motor unit potential; MUNE, motor unit number estimate; iMUNE, intramuscular motor unit number estimate; MUP, motor unit potential. P-values for significant differences are shown in bold.*

## Discussion

We describe here several novel observations: Firstly, MUP size was greater for athletic older men compared with younger athletes of the same discipline. MUP size was also greater for athletic old of both disciplines compared to non-athletic older men. Secondly, we show that older masters athletes have smaller CMAP and lower MUNE values for the VL than healthy younger men. MUNE values were similar for all groups of older men, regardless of their exercise discipline (control, endurance or power). Finally, the favored arm of masters tennis players had similar MUNE and iMUNE, but larger CMAP and MUP, compared with the non-favored arm. Taken together, these results suggest that for the VL and BB, long-term training does not prevent age-related decline in MU numbers, but may facilitate successful reinnervation of denervated fibers promoting an increase in MU size to preserve muscle function.

The athletes recruited to this study were nationally or internationally competitive. For example, the age-graded performance of young endurance athletes was 87%, equivalent to a 5 km run time of approximately 14:40 min. Their physical characteristics typified their competitive discipline; endurance athletes had the lowest body mass, fat and knee extensor MVC, while the young power athletes had relatively low body fat and the highest body mass, muscle mass and knee extensor MVC. The typical endurance characteristics were generally retained amongst older athletes, as we previously noted (Piasecki et al., 2018a).

The combination of type 2 fiber atrophy and loss of some type 1 and type 2 fibers determines the extent of muscle loss in older age (Lexell et al., 1988; McPhee et al., 2018; Wilkinson et al., 2018), which is associated with remodeling of MUs. A review of the available literature showed that healthy older limb muscles have approximately 40% fewer MUs and those that remain are around 30% larger than healthy young (Piasecki et al., 2016b). The extent of MU remodeling seems to influence overall muscle mass, as we previously showed that older sarcopenic individuals have approximately 50% fewer MUs compared with young and additionally, they fail to increase the average MU size (Piasecki et al., 2018c). One appealing hypothesis is that regular exercise throughout older age will help to retain MU numbers and by extension, muscle size and function. Some evidence to this effect was previously presented for the TA muscle (Power et al., 2010, 2016), but this was not replicated by our own past work (Piasecki et al., 2016a). Here, we show that VL CMAP and sMUP values were similar across all older groups (endurance, power and controls) and calculating MUNE values from these data suggests similar MU numbers across these older groups. In all cases, CMAP values were lower and sMUP values similar in old compared with young men.

A limitation of cross-sectional studies comparing young with old is that lifestyle and other secular changes might contribute to any observed differences. Akin to fiber number, it is probable that MU characteristics are also influenced by genetic factors (Degens and Korhonen, 2012). For these reasons, we included competitive masters tennis players to take advantage of discordant use of their arms to compare the localized effects of long-term exercise on MU characteristics. This study design minimizes influences of genetic and lifestyle factors. Here, MUNE values were similar for the BB of both arms, but the CMAP and the MUP size were larger in the BB of the favored arm.

Our results suggest that regular exercise does not preserve MU numbers into older age, but there are other possible benefits of training for MU plasticity. Regular exercise has been associated with possible improvements to fiber reinnervation in older age that would result in increased MU size and clustering of fibers of the same type, referred to as fiber type grouping (Mosole et al., 2014; Zampieri et al., 2015). Our work provides further direct evidence that MU size, estimated from the intramuscular MUP area, was larger in all groups of older compared to younger participants, which is in agreement with previous studies (Hourigan et al., 2015; Piasecki et al., 2016b). Importantly, age-related MUP size increases (assessed cross-sectionally) were greater for older endurance and power athletes than older controls (Figure 4), and were greater in the favored arm compared with the non-favored arm of masters tennis players (Table 3). Fiber reinnervation acts as a compensatory mechanism in response to MU loss (Gordon et al., 2004) and these results suggest lifelong exercise may facilitate this process. Although not a direct estimate of fiber number, the CMAP is an indicator of the amount of contractile material within the recording area of the surface electrode. This was larger in the favored compared to the non-favored arm of the tennis players, and although not significantly different, the VL CMAP was 8–15% larger in the older athletes compared to older controls. Thus, these data suggest the possibility of increased MU size in the master athletes and preserved muscle quality (Power et al., 2014; McPhee et al., 2018).

Regular intense exercise can preserve muscle mass and function in older age (Mckendry et al., 2018) by minimizing fiber atrophy and possibly by enhancing the rescue of denervated muscle fibers. It remains largely unknown how the local muscle milieu created by exercise leads to more successful rescue of orphaned fibers, but this will inevitably depend on facilitating successful axonal sprouting and remodeling of neuromuscular junctions (Deschenes, 2011; Gonzalez-Freire et al., 2014; Nishimune et al., 2014; Tintignac et al., 2015). However, caution should be shown when translating this evidence largely derived from animals into human aging (Jones et al., 2017).

### Strengths and Limitations

A strength of this work was the relatively large sample size and use of decomposition-enhanced quantitative electromyography to compare MU characteristics between groups of older and younger men, and the athletes enrolled in this study were of a high caliber. A second strength of this work was that in addition to the direct comparisons across age and exercise disciplines, we also assessed masters tennis players to take advantage of different bi-lateral use of limbs to increase confidence that effects were related to the exercise habits rather than other lifestyle or genetic characteristics.

A limitation of the work was that it is not possible to directly measure the number of MUs in human volunteers. We have used the iMUNE method to provide an index based on the mean MUP size normalized to the muscle CSA, and the more established method of comparing the electrically evoked CMAP to an ensemble-averaged mean sMUP (MUNE) (Brown et al., 1988; Piasecki et al., 2018b). Although limited by the capture area of the recording electrodes, results from both techniques were generally consistent across age; older adults have lower MU number values than young, irrespective of training history (Figure 4). A second limitation is that all MUs were sampled during isometric contractions held at 25% MVC, which precludes definitive statements on MU remodeling assessed across the full MU pool where smaller/larger MUs would be recruited in accordance with the Henneman size principle (Henneman et al., 1965). Thirdly, muscle biopsies were not collected in the present study, so it is not possible to estimate the contribution that fiber atrophy has on the age-related loss of muscle mass across the three disciplines, and whilst not affecting the conclusions of this study, it is likely that fiber atrophy occurred in all old groups. Muscle biopsies could also be used in future studies to assess for possible connective tissue (McPhee et al., 2018) and fat accumulation (Hogrel et al., 2015) with aging. Their accumulation may affect tissue conduction to attenuate the measured MUP (or similarly sMUPs and CMAP for surface recordings), which would mean that the increased MUP size of older men in the present study is a conservative estimation of the actual effect of aging. Finally, although the athletes were nationally or internationally competitive within their disciplines, we do not have sufficient detail of their training programs to compare training regimens across groups.

## Conclusion

Long-term athletic training does not prevent age-related decline of muscle size and the current findings suggest that MU numbers decrease even in athletic old. Long-term training into older age may facilitate more successful axonal sprouting and reinnervation of denervated fibers to produce larger MUs with increased MUP size.

## Ethics Statement

The study was conducted in accordance with the Declaration of Helsinki and approved by the University Research Ethics Committee and the National Research Ethics Service Committee Northwest (15/NW/0426). All participants provided written informed consent.

## Author Contributions

All authors contributed to the design of the work and analysis and interpretation of the data, and agreed to be accountable for all aspects of the work. MP, AI, JP, AS, and JM contributed to the acquisition of the data. MP and JM drafted the manuscript. AI, JP, HD, DS, AS, MR, and DJ provided comments and approved the final submission.

## Conflict of Interest Statement

The authors declare that the research was conducted in the absence of any commercial or financial relationships that could be construed as a potential conflict of interest.
